# Comparative Analysis of Dimensional Accuracy in PLA-Based 3D Printing: Effects of Key Printing Parameters and Related Variables

**DOI:** 10.3390/polym17121698

**Published:** 2025-06-18

**Authors:** Yifan Li, Amin Molazem, Hong-I Kuo, Vincent Ahmadi, V. Prasad Shastri

**Affiliations:** 1 Institute for Macromolecular Chemistry, University of Freiburg, 79104 Freiburg, Germany; yifan.li@makro.uni-freiburg.de (Y.L.);; 2 BIOSS Centre for Biological Signalling Studies, University of Freiburg, 79104 Freiburg, Germany

**Keywords:** additive manufacturing, fused deposition modeling, PLA, dimensional accuracy, printing parameters, Prusa MK4, LulzBot TAZ Pro

## Abstract

This study examines the impact of key printing parameters on the dimensional accuracy of 3D printing, specifically Fused Deposition Modeling (FDM) using PLA, utilizing two widely adopted printers: the LulzBot TAZ Pro and the Prusa MK4. A simplified parallel-line model was used to systematically evaluate the effects of print speed, nozzle temperature, bed temperature, and layer height on accuracy along the X, Y, and Z axes. The results showed that the Prusa MK4 generally provided better dimensional accuracy at lower print speeds (20–40 mm/s), higher nozzle temperatures (230 °C), and smaller layer heights (0.05 mm). In contrast, the LulzBot TAZ Pro performed better at higher print speeds (40–60 mm/s) and with thicker layers (0.2 mm). Scanning electron microscopy analysis further revealed distinct surface morphologies depending on the printer and parameter settings. These findings offer practical guidance for selecting suitable print settings across various application areas.

## 1. Introduction

The advent of three-dimensional printing (3DP) has fundamentally transformed the manufacturing landscape, enabling the production of complex geometries with a high degree of customization and precision, including labware and devices for biological research and clinical medicine [1,2,3,4]. This technology, which falls under the broader category of additive manufacturing (AM), represents a significant departure from traditional subtractive manufacturing methods. AM encompasses a range of technologies that build objects layer by layer from digital models, offering unprecedented flexibility in design and production. Among the various AM approaches, Fused Deposition Modeling (FDM), also known as Fused Filament Fabrication (FFF), stands out for its accessibility, cost-effectiveness, and versatility in material usage [5].

FDM has gained widespread popularity due to its ability to produce intricate shapes with minimal material waste, making it an ideal choice for rapid prototyping and the creation of customized parts. However, like all manufacturing technologies, it comes with its own set of challenges, including longer production times for larger components and the necessity for precise control over printing parameters to achieve high-quality outcomes [6]. Beyond FDM, other notable AM technologies include Stereolithography (SLA), Digital Light Processing (DLP), Selective Laser Sintering (SLS), and PolyJet printing, each with distinct advantages and limitations, often determined by the materials they can process and their specific applications [7]. The AM process starts with the creation of a digital 3D model using Computer-Aided Design (CAD) software, which is then converted into a printable format, such as Standard Tessellation Language (STL) file or Object File (OBJ), and processed by slicing software to generate Geometric Code (G-code) for layer-by-layer construction. In FDM, melted thermoplastic filament is extruded through a nozzle, solidifying layer by layer to form the object.

One of the most common materials used in FDM is poly (lactic acid) (PLA), a biodegradable, thermoplastic derived from renewable resources such as corn starch or sugarcane. PLA is favored not only for its perceived sustainability but also for its ease of use, making it particularly popular in educational settings, prototyping, and biomedical engineering [8]. Its relatively low melting temperature, typically ranging from 150 to 160 °C, allows it to be printed on a wide range of FDM machines without requiring a special printhead, reducing the risk of warping and ensuring consistent print quality. Furthermore, due to its solidification characteristics, PLA yields good dimensional accuracy, stability, and the ability to achieve a smooth surface finish, making it suitable for applications where precision and esthetics are paramount [4,9].

Achieving high dimensional accuracy in 3D-printed parts is crucial for ensuring their functionality and applicability in real-world scenarios. This is especially significant in the biomedical field, where precision directly impacts the performance and safety of devices used in clinical settings, including anatomical models used for diagnosis and surgical planning. In particular, the demands for dimensional accuracy are particularly high in the manufacturing of prostheses and medical devices, where patient-specific components must function as intended [2,3,10]. Inaccuracies in these scenarios can lead to parts that do not meet design specifications, potentially compromising the reliability and safety of the final product.

Recent studies have explored the relationships between various 3D printing parameters and their impact on dimensional accuracy. Researchers have investigated how printing speed, layer height, extrusion temperature, nozzle diameter, extrusion multiplier, and flow rate influence the accuracy of printed parts [11,12]. For example, Abas et al. [13] identified infill density as the most critical factor affecting length and width deviations in FDM-printed PLA parts, with layer height playing a significant role in angle and height deviations. Their findings suggest that a layer height of 0.1 mm, six perimeters, 20% infill density, a 90° fill angle, a print speed of 70 mm/s, a nozzle temperature of 220 °C, a bed temperature of 70 °C, and a 90° print orientation are an optimal set of printing parameters. Understanding and optimizing these parameters is crucial for achieving high precision and reproducibility in 3D printing. To further enhance precision, machine learning techniques have been employed to optimize printing parameters [14] in addition to exploring compounding, using inorganic fillers such as hydroxyapatite, to improve filament formation and solidification [15].

To date, there have been limited reports on the effects of a single (individual) factor on dimensional accuracy. Our study specifically focuses on how individual parameters such as printing speed, nozzle temperature, bed temperature, and layer height affect dimensional accuracy. By isolating these variables, we aimed to gain a deeper understanding of each factor’s unique contribution to the overall precision of the printed parts. To further understand the outcomes, we employed scanning electron microscopy (SEM) to analyze the extrusion width and microstructure of the printed samples. To make our findings broadly applicable, we first chose two open-source desktop printers that are widely used in research and biomedical laboratories. The Prusa MK4 and LulzBot TAZ Pro were selected because of their wide availability, open-source design, and frequent use in academic laboratories, prototyping, and biomedical settings. The Prusa MK4, a successor to the popular Prusa i3 series, is known for its reliable print quality, automatic bed leveling, and easy calibration, making it common in educational and research environments [16,17,18]. Likewise, the LulzBot TAZ Pro offers dual-extrusion, a broad material range, and a sturdy frame, making it suitable for larger or more complex prints [19,20]. Prior work has already shown their value in scaffold fabrication [21], anatomical modeling [22], and device prototyping [1], confirming their relevance to dimensional accuracy studies. This comparison allows us to assess the consistency and reliability of the dimensional accuracy across different print platforms, providing valuable insights for both academic research and practical applications in the field of 3D printing.

## 2. Results and Discussion

### 2.1. Influence of Printing Speed

In the dimensional accuracy of three-dimensional printed parts, printing speed plays a crucial role as it affects both the mechanics of filament deposition and the heat transfer in the printed structure. Lower speeds give each layer more time to dissipate heat before the next strand makes contact, promoting complete solidification, reducing shrinkage, and yielding tighter tolerances. This was most evident on the Prusa MK4, where the lowest *Z*-axis relative error was observed between print speeds of 20 and 40 mm/s. If the speed is reduced too far, residual nozzle heat can accumulate in small or discrete regions, softening previously deposited material and causing local sag or spread. Faster speeds invert the balance and lead to the deposition of a new layer onto a still-warm substrate, increasing thermal build-up. This can enhance interlayer adhesion but may also soften the underlying polymer, introducing *Z*-axis (height) errors and surface undulation (unevenness) unless the cooling is aggressive. The simulation and empirical data taken together suggest that a print speed of 40 mm/s is a window that promotes bonding, yet is slow enough to avoid steep thermal gradients [23].

**Figure 1:**
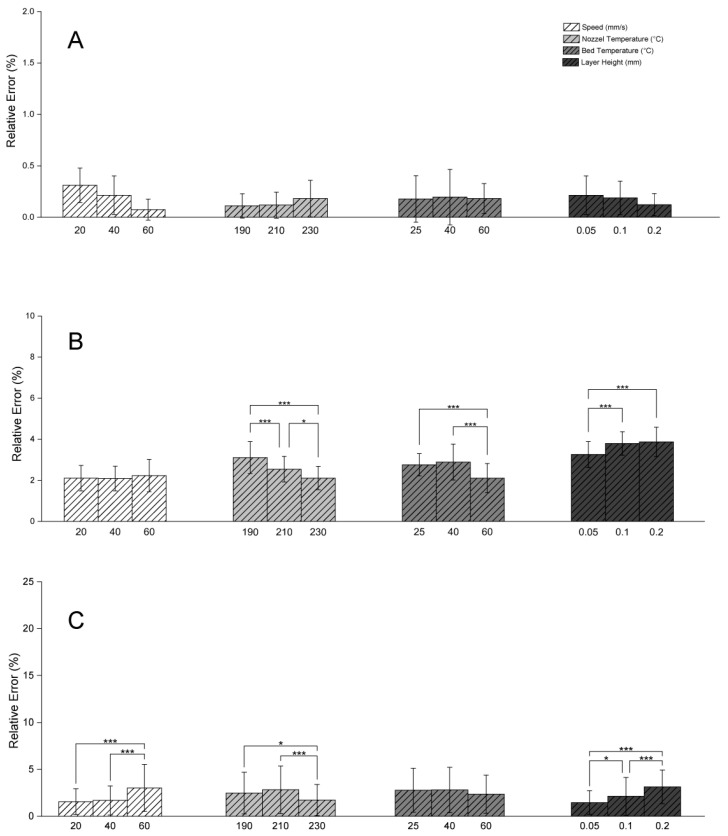
Relative dimensional errors in length, width, and height for Prusa MK4 under varying printing parameters (speed, nozzle temperature, bed temperature, layer height). (**A**) Relative error in length. (**B**) Relative error in width. (**C**) Relative error in height. * *p* ≤ 0.05, *** *p* ≤ 0.001.

**Figure 2:**
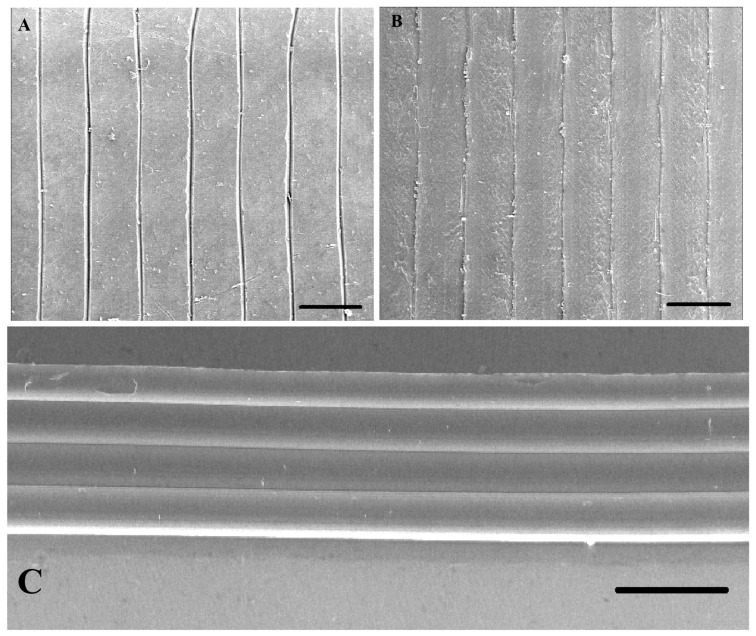
SEM images of objects printed using different 3D printers and speeds. (**A**) SEM image showing potential gaps in the sample printed using the LulzBot TAZ Pro printer at a speed of 20 mm/s. (**B**) SEM image of the sample printed using the Prusa MK4 printer at a printing speed of 60 mm/s. (**C**) SEM image illustrating the *Z*-axis height of the sample printed at a speed of 20 mm/s by Prusa MK4. Scale Bar = 500 µm.

At higher speeds, the printer experiences greater inertia and vibration, potentially leading to changes to the rigidity of the printer frame and reduced accuracy [12]. Moreover, high speeds limit the time available for the extruded filament to fully melt [24] and bond with previous layers and adjacent filaments [25]. Increasing the print speed also increases the shear stress in the polymer, causing the material to stretch more quickly, diminishing the filament’s width. Once the rate of shear crosses a certain threshold, extrusion swell (the polymer’s expansion post nozzle) diminishes [26,27]. In this study, in the Prusa MK4 printer, at higher speeds (such as 60 mm/s), greater errors in the vertical (Z) axis height were observed, while a more consistent placement of adjacent lines and even cooling is attained at lower speeds (20 mm/s or 40 mm/s) (Figure 1C and Figure 2C), minimizing deformation and gaps, and leading to better dimensional accuracy. The improved cooling at lower speeds is of consequence when printing PLA, as PLA requires sufficient time for each layer to solidify before the deposition of subsequent layers.

**Figure 3:**
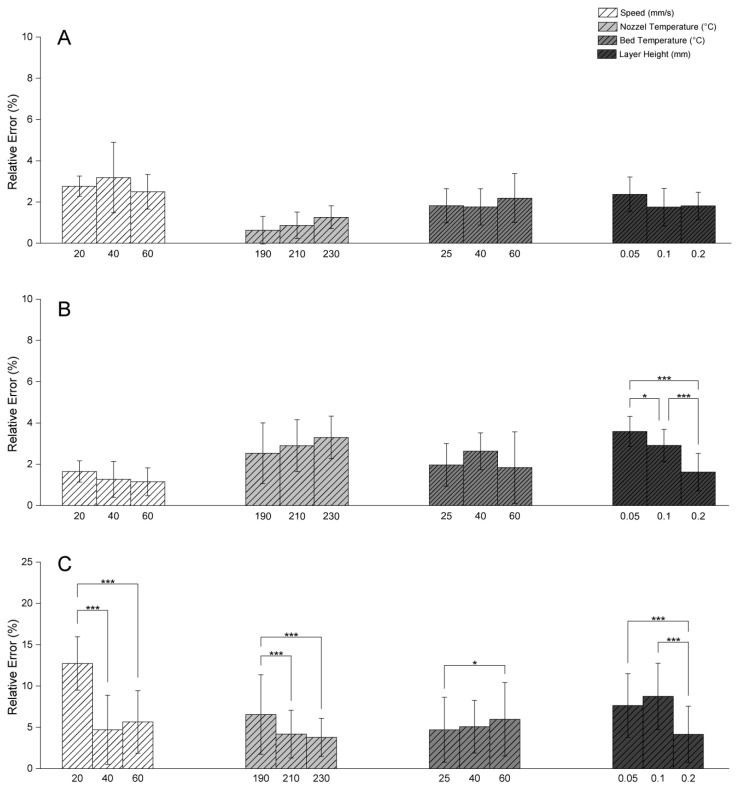
Relative dimensional errors in length, width, and height for LulzBot TAZ Pro under varying printing parameters (speed, nozzle temperature, bed temperature, layer height). (**A**) Relative error in length. (**B**) Relative error in width. (**C**) Relative error in height. * *p* ≤ 0.05, *** *p* ≤ 0.001.

**Figure 4:**
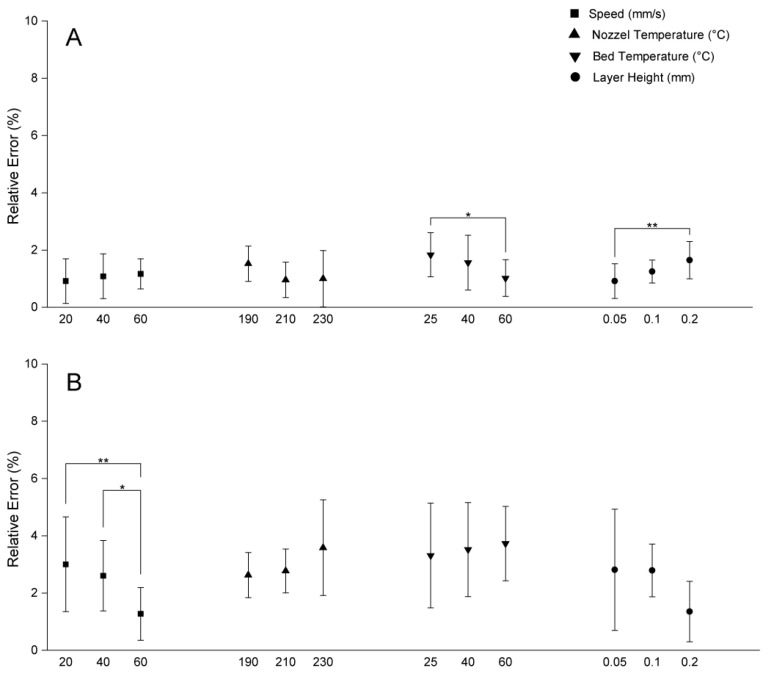
Relative dimensional errors in extrusion width for two printers under varying printing parameters (speed, nozzle temperature, bed temperature, layer height). (**A**) Relative error in extrusion width for Prusa MK4. (**B**) Relative error in extrusion width for LulzBot TAZ Pro. * *p* ≤ 0.05, ** *p* ≤ 0.01.

In addition to the accuracy of filament deposition, print speed can also impact the shape of the filament and lead to physical deformities. In this regard, slower print speeds can cause the material to overheat, sag, or spread, also leading to dimensional errors [13,28]. Buj-Corral et al. [28] found that increasing the speed from 30 mm/s to 40 mm/s reduced Z-direction dimensional errors, a finding echoed by Abas et al. [13] who reported that increasing the print speed from 50 mm/s to 70 mm/s cut dimensional deviation from 0.8% to 0.5%. However, slower speeds give the material more time to deposit, paradoxically increasing dimensional deviation due to polymer expansion [29]. In the case of the LulzBot TAZ Pro printer, a speed of 60 mm/s yielded the lowest relative error in both height and extrusion width (Figure 3C and Figure 4B). Statistical analysis revealed significant differences in extrusion width at various speeds, suggesting that higher speeds help mitigate issues such as localized overheating and excessive dwell time seen at slower speeds. In samples produced by the LulzBot TAZ Pro, lower speeds resulted in larger gaps between layers (Figure 2A), while a more consistent *Z*-axis height was achievable with the Prusa MK4 at the same print speed (Figure 2C). However, higher speeds in the Prusa MK4 led to more surface defects (Figure 2B). Hence, the effect of print speed on dimensional accuracy can be highly printer-dependent, influenced by factors such as frame stability, gear drive, and extrusion consistency.

### 2.2. Influence of Nozzle Temperature

**Figure 5:**
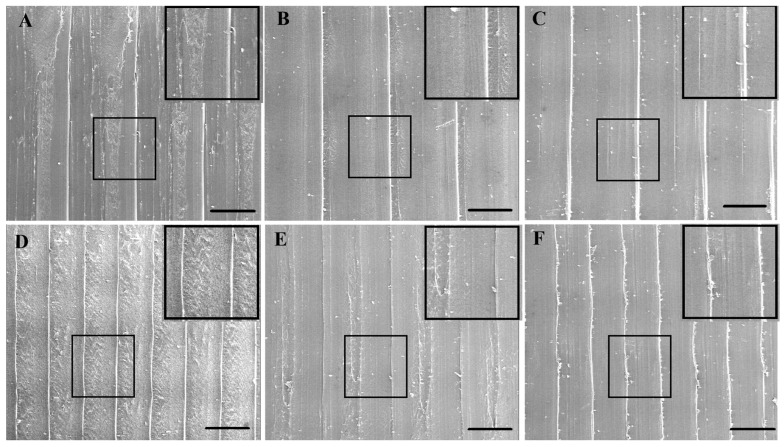
SEM images of printed Samples at different nozzle temperatures. (**A**) Sample printed with LulzBot TAZ Pro at a nozzle temperature of 190 °C, (**B**) 210 °C, and (**C**) 230 °C. (**D**) Sample printed with Prusa MK4 at a nozzle temperature of 190 °C, (**E**) 210 °C, and (**F**) 230 °C. The black square boxes indicate selected surface regions, which are shown magnified in the top-right insets for detailed observation of morphological features. Scale Bar = 500 µm.

The temperature of the nozzle has a noticeable impact on the viscosity of PLA during the extrusion process and consequently on the dimensional accuracy. Both the Prusa MK4 and LulzBot TAZ Pro printers generally showed better bonding quality in the X-Y plane at higher nozzle temperatures (210 or 230 °C), resulting in fewer gaps between adjacent extruded lines and more continuous deposition (Figure 5B,C,E,F). The decreased viscosity at higher temperatures promotes smoother material flow and more stable extrusion rates, leading to consistently deposited layers. However, lower temperatures resulted in poorly bonded layers, leading to uneven surfaces and reduced precision [30].

On the Prusa MK4, a nozzle temperature of 230 °C yielded lower relative errors in width and height (Figure 1B,C), demonstrating that higher temperatures help maintain precision. Nevertheless, at excessively high temperatures, minor surface irregularities or material deformation may occur due to overheating, as evidenced by the nominal surface irregularities in the Prusa MK4 samples printed at 230 °C.

For the LulzBot TAZ Pro, while a nozzle temperature of 230 °C also improved vertical (Z) axis accuracy (Figure 2C), a trend was observed in the *Y*-axis width, where lower temperatures resulted in a slightly reduced dimensional error compared to higher temperatures (Figure 2B), although not statistically significant. The increased likelihood of oozing at higher temperatures may contribute to greater width errors, as excess molten filament can be deposited unintentionally. This observation aligns with the findings of Beniak et al. [31] that an increase in nozzle temperatures can lead to greater dimensional errors due to enhanced flow from gravity and higher polymer expansion.

### 2.3. Influence of Bed Temperature

The bed temperature is also another parameter that influences initial layer adhesion and the solidification behavior of PLA. The T_g_ (glass transition temperature) of PLA is approximately 60 °C [32], and according to the manufacturer’s information, the T_g_ of the PLA filament used in this study is also around this value. When the bed temperature is set to near or above this threshold, the bottom layers remain softer for a longer period, increasing the risk of warping and dimensional distortions. In the LulzBot TAZ Pro printer, better *Z*-axis height accuracy was observed at bed temperatures of 25 °C and 40 °C, which is consistent with the improved solidification below the T_g_ (Figure 5C), and this promoted stable, rapid solidification of the first layers, minimizing curling or shrinkage at the corners. The Prusa MK4, in contrast, often yielded good outcomes in *Y*-axis width and extrusion width at a bed temperature of 60 °C (Figure 1B and Figure 3A). Although Abas et al. have reported an increase in deviation from prescribed dimensions with higher bed temperatures [13], likely due to the formation of more crystalline regions within PLA, which can cause additional shrinkage and internal stresses [33], one could also envisage another scenario where maintaining a bed temperature around 60 °C ensures adequate adhesion between printed PLA layers, which can also prevent excessive softening of the printed layers. Furthermore, rapid solidification of the initial layers can occur if the bed temperature is set too low, resulting in suboptimal adhesion and the development of internal stresses that can promote delamination [34].

### 2.4. Influence of Layer Height on Dimensional Errors

Layer height directly affects print resolution, surface finish, and potential accumulation of small errors across multiple layers [35]. When layer height is not accurately rendered, the nozzle may either land above the printed structure or impinge on it, both of which can cause print failure—the former by introducing discontinuities and the latter by inducing deformation. In the Prusa MK4 printer, smaller layer heights (0.05 mm) generally contributed to reduced relative errors in both width and height (Figure 1B,C). Thinner layers offer finer resolution [36], which is beneficial for capturing intricate details and minimizing the “stair-stepping” effect on inclined or curved surfaces [25,37]. Additionally, reducing the gap between layers can foster stronger interlayer adhesion, improving structural integrity and dimensional precision [13].

**Figure 6:**
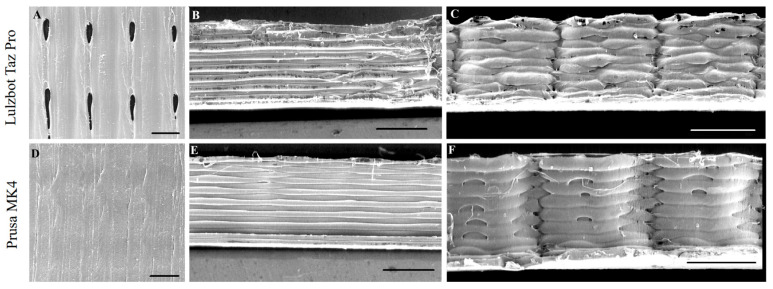
SEM images of printed samples with a layer height of 0.05 mm. (**A**–**C**) SEM images of the sample printed with the LulzBot TAZ Pro: (**A**) top view, (**B**) front view, (**C**) side view. (**D**–**F**) SEM images of the sample printed with the Prusa MK4: (**D**) top view, (**E**) front view, (**F**) side view.

However, smaller layer heights also increase total print time and may exacerbate issues such as oozing or stringing if the extruder’s filament flow is not meticulously controlled. For the LulzBot TAZ Pro printer, a thicker layer height (0.2 mm) often resulted in lower dimensional errors for the *Y*-axis width and *Z*-axis height (Figure 2B,C). Fewer overall layers mean that minor variations in layer deposition or nozzle positioning accumulate less prominently, leading to more consistent results in this particular printer setup [12]. Nevertheless, SEM images of LulzBot TAZ Pro structures printed at 0.05 mm layer height (Figure 6A–C) reveal notable defects, including oozing and gaps, stemming from the stringent precision requirements at fine resolutions. The absence of similar defects in objects printed using the Prusa MK4 under the same 0.05 mm layer height condition may be attributed to differences in hardware design and calibration systems between the two printers. The MK4 also appears to maintain more stable extrusion and layer placement at higher resolutions, likely due to a more responsive filament feeding mechanism, stronger localized cooling, and a more precise first layer calibration method. In contrast, the LulzBot TAZ Pro may be more sensitive to flow instability and thermal effects at such thin layers, which can lead to minor defects such as oozing or small gaps, as observed in Figure 6A–C. These performance differences highlight how printer-specific mechanical and control system characteristics can influence print quality, especially under demanding resolution conditions.

### 2.5. Comparison of Dimensional Accuracy Between the Two 3D Printers

**Figure 7:**
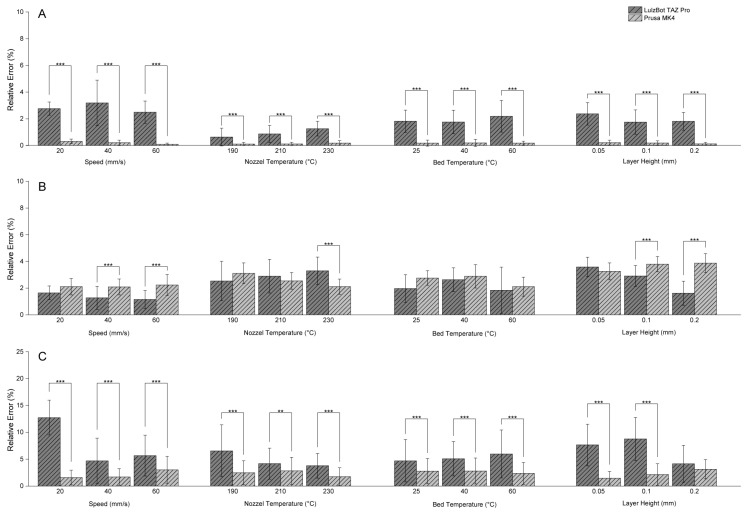
Relative dimensional errors of two 3D printers under varying printing parameters. (**A**) Relative error in length, (**B**) relative error in width, (**C**) relative error in height. ** *p* ≤ 0.01, *** *p* ≤ 0.001.

Overall, the Prusa MK4 printer exhibited lower relative errors in length (Figure 7A) and height (Figure 7C) for most parameter variations, including print speed, nozzle temperature, bed temperature, and layer height. Yet, in terms of the width of the print, at 40 and 60 mm/s or layer heights of 0.1 and 0.2 mm, the LulzBot TAZ Pro sometimes outperformed the Prusa MK4 (Figure 7B). These differences underscore the importance of matching printer characteristics with the process parameters to achieve optimal dimensional accuracy.

SEM observations (Figure 4, Figure 5 and Figure 6) further revealed that the LulzBot TAZ Pro samples occasionally displayed gaps between layers, suggesting less consistent extrusion under certain conditions. By contrast, the Prusa MK4 tended to form more uniform layers, benefiting from efficient firmware optimization, mechanical stability, and robust cooling.

In many medical applications, such as surgical guides for procedures in orthopedics, spine surgery, or total knee arthroplasty, high levels of detail and dimensional accuracy are important [2,3,4]. The Prusa MK4 printer, especially at lower layer heights (for example, 0.1 mm or 0.05 mm) and moderate speeds (20 mm/s or 40 mm/s), demonstrated superior performance for tasks requiring exact reproductions of intricate features, which is relevant in biomedical device fabrication.

For larger models or applications where fine details are less critical—such as prosthetics, orthotics [38], or frameworks for Otorhinolaryngology (E.N.T.) or plastic surgery [39]—the LulzBot TAZ Pro printer set at higher speeds (40 mm/s or 60 mm/s), higher layer heights (around 0.2 mm), and appropriate nozzle temperatures can offer efficient production while maintaining acceptable dimensional accuracy [40]. The printer’s ability to quickly produce robust parts with relatively low dimensional error makes it a practical choice for these scenarios.

## 3. Materials and Methods

### 3.1. Sample Preparation

The 3D printing process began with the design phase, which was carried out using Autodesk Fusion 360 (260.1.25, Autodesk Inc., San Francisco, CA, USA). The designed model was then processed for 3D printing using Simplify3D software (5.1.2, Cincinnati, OH, USA) for slicing, converting the model into machine-readable instructions. Two 3D printers, both equipped with a 0.4 mm nozzle, were utilized in this study: the Prusa MK4 (Prague, Czech Republic) and the LulzBot TAZ Pro (Fargo, ND, USA). Detailed configurations for each printer are presented in Table 1. For filament, PLA (Polymaker PolyLite, Changshu, China) was employed, with specific configurations detailed in Table 2. The printing parameters were as follows: print speed ranged from 30 to 60 mm/s, nozzle temperature varied between 190 and 230 °C, bed temperature ranged from 25 to 60 °C, and layer height was between 0.05 and 0.2 mm. Details of the parameter design specifications can be found in Table 3 and Table 4. All samples were printed with 100% infill and aligned according to a consistent pattern.

### 3.2. Measurement of Dimensional Accuracy

**Figure 8:**
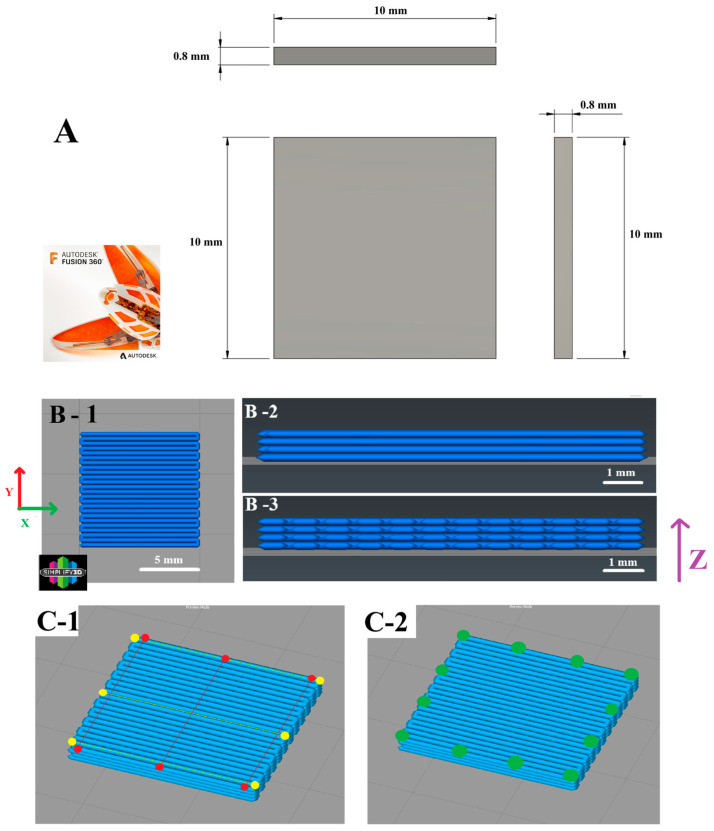
Workflow for this study: starting with object design, slicing, SEM imaging, and measurement of the sample dimensions. (**A**) CAD created using Fusion 360. (**B**) Sliced model: (**1**) Top view, (**2**) front view, (**3**) side view. The Y direction represents the width, the X direction represents the length, and the Z-axis represents the height. (**C**) Measurement locations: (**1**) The lines between the yellow points indicate the positions used for measuring length, while the lines between the red points indicate the positions used to measure width. (**2**) A total of 12 spots were measured to determine the thickness, referred to as “height” in the paper.

A total of 9 specimens were printed for each parameter set. The specimens were designed as simple squares, each measuring 10 × 10 mm with a height of 0.8 mm (Figure 8A). The definition and orientation of the X, Y, and Z axes of the specimen, including top and side views, are illustrated in Figure 8B. The methods and locations for measuring the length, width, and height of the samples are illustrated in Figure 8C. In this study, the curved side of the specimen is defined as the *Y*-axis width, while the linear portion is defined as the *X*-axis length. For each sample, both the width and length were measured three times from lateral to medial. For *Z*-axis height measurements, each side of the specimen was also measured three times from lateral to medial, resulting in a total of 12 measurements per sample. Dimensional accuracy was measured using digital calipers (Bahag AG, Mannheim, Germany), which provide a resolution of 0.01 mm and an accuracy of ±0.02 mm for measurements under 100 mm and ±0.03 mm for those over 100 mm. Additionally, the extrusion width was measured by SEM, and three samples with different parameters were randomly selected for analysis. Measurements were taken at the 1/3 and 2/3 width positions of two randomly chosen extrudes, and the results were recorded accordingly. The printing error was calculated using the following formula:

$$\mathrm{Relative}\;\mathrm{Error}\;\left(\%\right)=\frac{\mathrm{Measured}\;\mathrm{Value}-\mathrm{Prescribed}\;\mathrm{Value}}{\mathrm{Prescribed}\;\mathrm{Value}\;(\mathrm{Real}\;*)}\times 100$$

### 3.3. Scanning Electron Microscopy

The extrusion width and sample morphology were analyzed using a FEI Quanta 250 FEG SEM. The SEM was operated with an accelerating voltage of 20 kV. To enhance surface detail, the secondary electron mode was utilized. The samples were coated with a thin layer of gold (Au) for 90 s to ensure electrical conductivity and minimize charging during imaging. The chamber pressure was maintained at 100 Pa during scanning. Images were acquired at various magnifications, providing both an overview and a detailed examination of surface features.

### 3.4. Statistical Analysis

Statistical analysis was performed using OriginPro 2024 SR1 (OriginLab Corporation, Northampton, MA, USA). One-way analysis of variance (ANOVA) was used to compare differences within groups, followed by pairwise comparisons using the Tukey test. A *p*-value of <0.05 was considered statistically significant.

## 4. Conclusions

The objective of this effort was to ascertain the limitations of the printing platforms and determine their suitability for printing precision structures. The effect of printing process parameters (print speed, nozzle temperature, bed temperature, and layer height) on the fused filament modeling of PLA using two commonly used printers, namely, the LulzBot TAZ Pro and Prusa MK4, was investigated using a simple model of an array of parallel lines. The influence of printing speed, nozzle temperature, print bed temperature, and layer height on dimensional accuracy was quantified by examining the printed parts using scanning electron microscopy. Using this quantitative analysis, printer-specific optimal conditions were identified. Among the process variables investigated, layer height was the most significant factor impacting dimensional accuracy. Adjusting layer height caused the largest and most consistent shifts in both width and height errors. A thinner layer raised vertical resolution and increased the number of layers, which tightened *Z*-axis precision, reducing the “stair-step” error that accumulates in the X-Y plane. Thinner layers also bonded more reliably and lowered the occurrence of layer drifting off line, so overall tolerances were improved. Conversely, thicker layers (e.g., 0.20 mm), while shortening print time as expected, can aid in steadier flow of material and heat removal, which, depending on the machine, may reduce errors in simple parts. Print speed and nozzle temperature mainly influenced accuracy through heat build-up and flow stability, and their effects varied widely between the two printers. The optimal layer height was therefore printer-specific, with 0.05 mm layer height yielding the smallest errors on the Prusa MK4, while a 0.20 mm layer height worked best on the LulzBot TAZ Pro. The Prusa MK4 delivered the highest precision at lower speeds and smaller layer heights, while the LulzBot TAZ Pro performed better at higher speeds and thicker layers. Users must therefore consider trade-offs between precision in parts and build time, since halving the layer height roughly doubles the number of layers and extends printing time accordingly. The results from this study highlight the practical performance limits of the two printers and provide guidelines for print settings that balance speed and accuracy, which are especially important in biomedical or mechanical prototyping. Furthermore, the approach used in this study has applicability in a broader context of material-specific and printer-specific process optimization and helps establish standard practices in manufacturing using FDM.

## Figures and Tables

**Table 1:** Specifications of FDM 3D printers.

	FDM 3D Printer	
Manufacturer	Lulzbot TAZ Pro *	Prusa MK4 **
	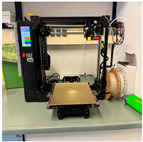	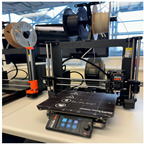
Print Area	280 mm × 280 mm × 285 mm	250 × 210 × 220 mm
Print Volume	22.344 L	11.550 L
Layer Resolution	0.05 mm–0.4 mm	0.05 mm—0.3 mm
Filament Diameter	2.85 mm	1.75 mm
Type’s of Extrusion	Direct Drive	Direct Drive
Print Bed	Not Removeable Borosilicate Glass with PEI Surface	Removeable Smooth PEI Print Sheet
Calibration	Automatic	Automatic Mesh Bed Leveling
Max Nozzle Temperature	290 °C	290 °C
Max Heat Bed Temperature	100 °C	120 °C
Nozzle Material	Brass—V6 Extra E3D	Brass—V6

* LulzBot Taz Pro, Fargo, North Dakota; ** Prusa Research a.s. EU MWSt. Nummer: CZ06649114 Partyzánská 188/7A 17,000 Prag 7 Tschechische Republik.

**Table 2:** Properties and recommended print settings for Polymaker PolyLite™ PLA.

Polymaker Polylite PLA *			Recommended Print Settings
Nozzle Temperature	190–230	°C	
Bed Temperature	25–60	°C	
Nozzle Speed	40–60	mm/s	
Cooling Fan	On	-	
Density	1.17–1.24	g/cm^3^ @21.5 °C	ASTM D792
Glass transition Temperature	61	°C	DSC, 10 °C/min
Melt Index	7–11	g/10 min	210 °C, 216 kg
Melting Temperature	150	°C	DSC, 10 °C/min
Crystallization Temperature	114	°C	DSC, 10 °C/min
Young’s Modulus (X-Y)	2636 ± 330	MPa	ASTM D638
Tensile Strength (X-Y)	46.6 ± 0.9	MPa	ASTM D638

* PolyLite™ PLA—Polymaker, Changshu City, Jiangsu Province, China.

**Table 3:** Printing parameters for LulzBot TAZ Pro and Prusa MK4 (constant parameters).

Device	LulzBot Taz Pro	Prusa MK4	Unit
Extruder	Right	Main	-
Nozzle Diameter	0.4	0.4	mm
Extrusion Multiplier	1	1	-
Retract Distance	1	1	mm
Retract Speed	10	10	mm/s
Top Solid Layer	0	0	-
Bottom Solid Layer	0	0	-
Outline Perimeters	0	0	-
Internal Infill Pattern	Aligned	Aligned	-
Infill Percentage	100	100	%
Infill Extrusion Width	Extrusion Width	Extrusion Width	mm
Cooling First Layer	0	0	%
Cooling from 2nd Layer	100	100	%
3D Printing Adhesive	Magigoo * Universal	Magigoo Universal	-

* Magigoo, Thought3D Ltd., Unit2150, KBIC, Kordin Industrial Estate, Paola, PLA3000, Malta.

**Table 4:** Printing parameters for LulzBot TAZ Pro and Prusa MK4 (variable parameters).

	Speed Test	NT Test	BT Test	LH Test	Unite
Speed	20/40/60	40	40	40	mm/s
NT	210	190/210/230	210	210	°C
BT	40	40	25/40/60	40	°C
LH	0.2	0.2	0.2	0.05/0.1/0.2	mm

NT: Nozzle Temperature, BT: Bed Temperature, LH: Layer Height.

## Data Availability

The original contributions presented in the study are included in the article; further inquiries can be directed to the corresponding author.
